# Rapid antibiotic susceptibility testing from blood culture bottles with species agnostic real-time polymerase chain reaction

**DOI:** 10.1371/journal.pone.0209042

**Published:** 2018-12-13

**Authors:** Tucker Maxson, Candace D. Blancett, Amanda S. Graham, Christopher P. Stefan, Timothy D. Minogue

**Affiliations:** Diagnostic Systems Division, United States Army Medical Research Institute of Infectious Diseases, Fort Detrick, Maryland, United States of America; University of Malaya, MALAYSIA

## Abstract

Development and implementation of rapid antimicrobial susceptibility testing is critical for guiding patient care and improving clinical outcomes, especially in cases of sepsis. One approach to reduce the time-to-answer for antimicrobial susceptibility is monitoring the inhibition of DNA production, as differences in DNA concentrations are more quickly impacted compared to optical density changes in traditional antimicrobial susceptibility testing. Here, we use real-time PCR to rapidly determine antimicrobial susceptibility after short incubations with antibiotic. Application of this assay to a collection of 144 isolates in mock blood culture, covering medically relevant pathogens displaying high rates of resistance, provided susceptibility data in under 4 hours. This assay provided categorical agreement with a reference method in 96.3% of cases across all species. Sequencing of a subset of PCR amplicons showed accurate genus level identification. Overall, implementation of this method could provide accurate susceptibility results with a reduced time-to-answer for a number of medically relevant bacteria commonly isolated from blood culture.

## Introduction

Introduction of antibiotics into patient treatment strategies drastically reduced bacterial infection related morbidity and mortality and opened the door to medical procedures that otherwise carry high risk of infection, including invasive surgeries and medical device implantation. Unfortunately, the spread of antimicrobial resistance and the emergence of multi-drug resistant strains is a global problem limiting treatment options and contributing to prescribing of ineffective antibiotic regimens that fail to resolve infections [1]. Therefore, it is imperative for clinicians to have access to susceptibility data when prescribing antimicrobial agents to avoid the empirical selection of ineffective treatments, especially in life-threatening, time sensitive cases such as sepsis [2, 3]. Traditional, culture-based antimicrobial susceptibility testing (AST) often takes more than 48 h for time-to-answer [4], an inappropriate timeframe for guiding early treatment strategies; thus, development of more rapid AST assays could potentially mitigate these issues [5, 6]. A further benefit of rapid AST is the fostering of the initiative towards antimicrobial stewardship, which aims to reduce unnecessary antibiotic usage to improve patient outcomes and reduce selection-induced antimicrobial resistance [7, 8]. Rapid evolution and propagation of resistance determinants coupled with the challenges of developing novel antimicrobial agents and treatment methods highlights the importance of maintaining the effectiveness of currently available antimicrobial drugs [9].

An ideal AST assay would report phenotypic susceptibility, be applicable across a broad range of human pathogens and antibiotics, function with clinical samples, and deliver a result in the shortest amount of time possible. A variety of novel approaches work towards these goals, including but not limited to: genotyping [10, 11], single cell microscopy [12, 13], Raman spectroscopy [14], mass spectrometry [15–17], and nucleic acid amplification [18–22]. Each of these strategies has strengths and weaknesses, and ongoing work within respective fields continues to push each methodology towards the ideal AST. In particular, strategies focusing on nucleic acid amplification are especially appealing as DNA and RNA cellular synthesis can be very rapid and is proportionally impacted either directly or indirectly by antibiotic inhibition of susceptible cells.

In general, nucleic acid amplification strategies for AST compare relative quantification of DNA or RNA concentrations between antibiotic-treated samples and untreated controls post-antibiotic treatment. Bacterial strains susceptible to the given antibiotic will display growth inhibition resulting in significantly lower concentrations of nucleic acid, measurable by quantitative PCR (qPCR) or other amplification technologies [18]. Previous studies utilizing this approach showed promising results with high levels of categorical agreement with gold standard assays [18–21]. In fact, the method adequately determined susceptibility across multiple classes of antibiotics and was applicable to a variety of bacterial pathogens depending on the primer (and probe) design [19, 21]. More recent studies focused on reducing the overall assay time-to-answer, with specific focus on rapid results obtained through the use of isothermal amplification technologies [20].

The present study aims to assess a previously reported, broad-coverage qPCR primer and probe set, the BactQuant assay, for use in AST assay development [23]. The BactQuant assay targets the V3-V4 region of the 16S gene sequence with specific design features providing improved genus and species coverage over earlier 16S-targeting assays [23]. Here, we modified the method for the RApid Molecular Antibiotic Susceptibility Testing (RAMAST) assay reported by Beuving et al. [19] to reduce the time-to-answer and increase species coverage and evaluated the assay with a large set of important human pathogens. The RAMAST assay was performed on mock blood cultures with three disparate antibiotics per species, demonstrating the applicability of this assay to multiple antibiotic classes.

## Materials and methods

### Bacterial isolates and culture

Clinical isolates and reference strains were obtained from the American Type Culture Collection (ATCC), the Biodefense and Emerging Infections Research Resources Repository (BEI Resources), the Food and Drug Administration (FDA), the Centers for Disease Control (CDC), and the Unified Culture Collection housed at United States Army Medical Research Institute of Infectious Diseases (USAMRIID). The origin of each strain is listed in Tables A-E in S1 File. Isolates were propagated on blood agar (trypticase soy agar with 5% sheep blood) at 35°C and cultured in tryptic soy broth (TSB) liquid medium at 37°C with shaking unless otherwise noted. *Staphylococcus aureus* 880 (BR-VRSA) was found to rapidly lose vancomycin resistance in liquid culture and was thus propagated in the presence of 32 μg/mL vancomycin when liquid culture was required; cultures with vancomycin were then diluted at least 1,000 fold before use in any assay (32 ng/mL is well below the vancomycin minimum inhibitory concentration (MIC) of all strains tested). Antibiotics were obtained from Sigma-Aldrich, Thermo Fisher Scientific, or Gold Biotechnology and used at the concentrations given from 100x stock solutions in water.

### Gold standard antibiotic susceptibility testing

Reference MICs were determined by the broth microdilution (BMD) method in accordance with Clinical and Laboratory Standards Institute (CLSI) guidelines [24]. *Enterococcus faecium* NR-32094 did not display visible growth in the standard testing media (cation adjusted Mueller Hinton broth, CAMHB) and was tested in TSB instead. *Staphylococcus aureus* ATCC 13565 did not display visible growth in the presence of additional 2% NaCl and was tested without the added NaCl for oxacillin. The susceptibility breakpoints for antibiotics were taken from the 2017 CLSI guidelines [25].

### Assessment of antibiotic susceptibility using qPCR

Bacterial cultures prepared in TSB media were inoculated from colonies on fresh blood agar plates and were incubated at 37°C with shaking overnight (~16 h). The overnight cultures were used to spike 10 mL of human whole blood (BioIVT, Maryland, USA), and the entire aliquots of blood were injected into BD BACTEC standard/10 aerobic/F bottles (Becton Dickinson, New Jersey, USA). Blood culture (BC) bottles were incubated in a BD BACTEC FX40 instrument until flagged as positive by the instrument software. Within 30 min of positivity, 500 μL of culture was removed from each bottle and diluted into 4.5 mL of room temperature TSB. This diluted culture was then used to make 200 μL aliquots. Either antibiotics at the concentrations to be tested or no-antibiotic controls were added to each aliquot from 100x stocks in water. The aliquots were vortexed briefly and then incubated at 37°C with shaking in a ThermoMixer (Eppendorf, Hamburg, Germany) for 1 or 2 h. After incubation, samples were centrifuged at 16,000 x g for 3 min at room temperature and the supernatant was carefully removed by pipet.

Pellets were resuspended in 200 μL lysis buffer (10 mM Tris-HCl, 1 mM disodium EDTA, pH 8.0, 20 mg/mL lysozyme, 300 U/mL mutanolysin, 1.2% Triton X-100) and incubated at 37°C with shaking at 1,200 rpm in a ThermoMixer for 20 min. Glass beads (0.1 mm PowerBeads; Qiagen, Hilden, Germany) were then added to the samples to an approximate volume of 20 μL and the samples were vortexed on the fastest setting for 5 min. Buffers and spin columns from a QIaAMP DNA Mini Kit (Qiagen) were then used to complete the DNA extraction. To the bead beat samples, 200 μL of buffer AL was added and the samples were mixed by brief vortexing. Then, 200 μL of 100% ethanol was added and the samples were again mixed by brief vortexing. The samples were centrifuged briefly and 450 μL of supernatant was transferred to the kit spin columns. The samples were then washed according to the manufacturer’s instructions and eluted in 200 μL of water.

Eluted DNA was diluted 100 fold in water before use in qPCR. The qPCR was performed as previously described, with slight modifications [23]. Briefly, the PCR reactions were run in 10 μL volumes and were composed of the universal 16S BactQuant primers (5’-CCTACGGGDGGCWGCA-3’ and 5’-GGACTACHVGGGTMTCTAATC-3’) at 1.8 μM, probe (6FAM-5′-CAGCAGCCGCGGTA-3′-MGBNFQ) at 0.2 μM, Platinum qPCR Supermix-UDG (ThermoFisher) at 1x, water, and diluted sample DNA (2.5 μL). The amplification was performed on a LightCycler 480 II instrument (Roche, Basel, Switzerland) with cycling conditions of 50°C for 2 min, 95°C for 2 min, and 40 cycles of 95°C for 15 s and 60°C for 45 s. Quantification cycle (Cq) values were calculated automatically with the second derivative max method. The qPCR for each sample was run as technical triplicates, and the three values were averaged to give the final Cq value used in data analysis.

### Data analysis

To determine antibiotic susceptibility, ΔCq values were calculated by subtracting the Cq value obtained from the no-antibiotic control sample from the Cq values for the antibiotic-treated samples. Cutoff ΔCq values were determined for each species (or family for *Enterobacteriaceae* spp.) from ROC curves derived from the ΔCq values determined for each isolate (Figure A in S1 File). Cutoff values for each species were selected that maximized the likelihood ratio for a correct susceptibility call. ΔCq values used as cutoffs for each species or family were: > 2.25 for *Enterobacteriaceae* spp.; > 2.00 for *Acinetobacter baumannii*; > 1.70 for *Pseudomonas aeruginosa*; > 1.55 for *Enterococcus faecium*; and > 2.05 for *S*. *aureus*. A ΔCq value above the cutoff was used to indicate susceptibility to the antibiotic while a ΔCq value below the cutoff indicated resistance. Errors were defined as minor, major, or very major. Major errors were defined as false-resistant results and the major error rate was calculated as the number of false-resistant results over the total number of susceptible isolates as determined by BMD. Very major errors were defined as false-susceptible results and the very major error rate was calculated as the number of false-susceptible results over the total number of resistant isolates as determined by BMD. Strains with an intermediate resistance result from BMD were treated as resistant for the purposes of calculating categorical agreement, and a susceptible result from the qPCR assay for an intermediate strain was defined as a minor error.

### Amplicon sequencing and analysis

Amplicons from the qPCR assay were sequenced with a MiSeq system (Illumina, California, USA). Amplicons were first run through automated PCR purification using an Apollo 324 NGS Library Prep System (Takara Bio, Kusatsu, Japan) running the instrument’s “PCR Cleanup 32” protocol. TruSeq HT adaptors (Illumina) were then ligated using the Apollo 324’s “PrepX ILM 32i DNA” protocol for 520 base pair size, utilizing a SMARTer PrepX Universal DNA Library kit (Takara Bio). PCR enrichment was performed on the library products by adding 25 μL of KAPA HiFi HotStart ReadyMix (Roche), 5 μL of KAPA Library Amplification Primer Mix, and 5 μL of molecular biology grade water to each sample followed by a thermal cycling program of 98°C for 45 s, 10 cycles of [98°C for 15 s, 60°C for 30 s, and 72°C for 30 s], and 72°C for 1 min. PCR purification was performed again as above before DNA was quantified, diluted, and pooled. Pooled DNA was quantified by qPCR using a KAPA Library Quantification Kit (Roche), then further diluted to 2 nM. The 2 nM pooled DNA library was mixed with an equal volume of 0.1 M NaOH and incubated at room temperature for 5 min to denature. A PhiX library was also prepared by mixing equal volumes of 2 nM PhiX Sequencing Control v3 (Illumina) with 0.1 M NaOH and incubating at room temperature for 5 min to denature. Both denatured libraries were diluted to 12 pM with pre-chilled buffer HT1 (from a MiSeq Reagent kit v2 (Illumina)), pooled to give a final ratio of 25% PhiX, and sequenced on a MiSeq with a 600-cycle MiSeq cartridge with paired-end reads.

Analysis was performed using CLC Genomics Workbench (Qiagen). Imported paired end reads were merged and trimmed using a quality score 0.05 and a sequence cutoff length of >100 base pairs. The Ribosomal Database Project (RDP) was used to curate a down selected reference database composed of isolates of type strains from genera of medically relevant pathogens (with the inclusion of all species in those genera) as previously reported [26]. Only entries of greater than 1,200 base pairs with good quality were used in the curated database. A stringent reference-based mapping of sequencing reads to the curated RDP was used with mapping settings as follows: mismatch cost of 10, insertion cost of 3, deletion cost of 3, insertion open cost of 6, insertion extend cost of 1, deletion open cost of 6, deletion extend cost of 1, length fraction of 0.5, and similarity fraction of 1. Non-template controls were used to account for sample bleed and a cutoff was calculated using the mean plus 3 times the standard deviation for each genus. Reads which fell below this cutoff were removed and total reads mapped for each sample were calculated along with the representative percentage of each genus in the sample.

## Results and discussion

### Assay design and optimization

We chose to perform the RAMAST assay on extracted DNA due to the necessity for determining relative bacterial growth through quantitative DNA concentration measurements. While the addition of a DNA extraction before qPCR increased the turn-around-time of the assay, this step decreased the likelihood of qPCR inhibition by culture components for establishing a proof of concept for this assay. Ensuring the removal of PCR inhibitors is especially important when blood is present in a sample [27], as was the case in this study. Previous work examining AST by qPCR primarily utilized urine samples or pure culture, with one exception which used bacteria from blood culture bottles partially purified with serum separator tubes before the incubation with antibiotics [19]. Because initial testing with the standard lysis procedure stipulated in the DNA extraction kit protocol did not appear to result in complete lysis, especially for dense cultures of Gram-positive strains, our DNA extraction method included a rigorous cell lysis procedure including enzymatic digestion and bead beating to ensure complete lysis (Figure B in S1 File). Moving forward, alternative sample preparation procedures prior to qPCR could be utilized to decrease the assay time while maintaining adequate performance, particularly for Gram-negative species.

### Susceptibility testing on positive blood cultures

Empirical assessment is required before any assay can be matured through clinical evaluations. Towards this end, we tested a total of 144 clinical and reference isolates after mock blood culture for antibiotic susceptibility with the RAMAST assay: 50 *Enterobacteriaceae* spp., 30 *Acinetobacter baumannii*, 22 *Pseudomonas aeruginosa*, 22 *Enterococcus faecium*, and 20 *S*. *aureus* (Table 1). Assay optimization for three antibiotics per species (ciprofloxacin, gentamicin, and imipenem for Gram-negatives; ciprofloxacin, vancomycin, and oxacillin for *S*. *aureus*; ciprofloxacin, vancomycin, and ampicillin for *E*. *faecium*), across multiple concentrations spanning CLSI breakpoints determined the optimal concentration for distinguishing susceptible from resistant strains. Optimal concentrations are found for each species-antibiotic combination in Table 1. Due to the need for different antibiotic concentrations for different species, the assay is best performed after species identification. Temporally, we found an antibiotic incubation time of 2 h to be sufficient across all antibiotics with the exception of ciprofloxacin, which only required a 1 h antibiotic incubation. Shorter incubation times required for ciprofloxacin may be due to the fluoroquinolone antimicrobial inhibitory mode of action, which involves inhibition of DNA gyrase and topoisomerase IV activity through formation of drug-enzyme-DNA complexes with indirect consequences for DNA replication [28]. Inhibition of gyrase activity results in double-strand DNA breaks in metabolically active bacteria, terminating further DNA replication likely with higher rapidity than possible with other antibiotic classes. Other DNA targeting antimicrobials could also afford a shorter required incubation time, as noted in the literature for both nitrofurantoin and ciprofloxacin [20].

**Table 1:** Number of tested isolates and the antibiotic concentration used in the qPCR assay.

*Enterobacteriaceae* spp. (N = 50)		Susceptible*	Intermediate*	Resistant*	Antibiotic conc. tested
	Ciprofloxacin	23	0	27	2 μg/mL
	Gentamicin	31	2	17	16 μg/mL
	Imipenem	24	1	25	4 μg/mL
***A*. *baumannii* (N = 30)**					
	Ciprofloxacin	5	0	25	2 μg/mL
	Gentamicin	7	3	20	8 μg/mL
	Imipenem	16	1	13	4 μg/mL
***P*. *aeruginosa* (N = 22)**					
	Ciprofloxacin	5	0	17	2 μg/mL
	Gentamicin	5	0	17	8 μg/mL
	Imipenem	8	1	13	2 μg/mL
***E*. *faecium* (N = 22)**					
	Ciprofloxacin	7	3	12	1 μg/mL
	Vancomycin	14	0	8	1 μg/mL
	Ampicillin	10	0	12	16 μg/mL
***S*. *aureus* (N = 20)**					
	Ciprofloxacin	11	0	9	2 μg/mL
	Vancomycin	18	0	2	2 μg/mL
	Oxacillin	8	0	12	0.5 μg/mL

* Categorical assignment from reference method (BMD)

Ciprofloxacin is not generally indicated for blood stream infections with *E*. *faecium* and *S*. *aureus* [24]; however, we included this fluoroquinolone to highlight the shorter incubation time. In addition, this assay could be easily repurposed to different matrices, such as urine, or bacterial species where testing ciprofloxacin would be more appropriate. With the optimized incubation times, the overall assay turn-around-time was approximately 3 h for ciprofloxacin and 4 h for the other antibiotics post blood culture positivity.

**Fig 1:**
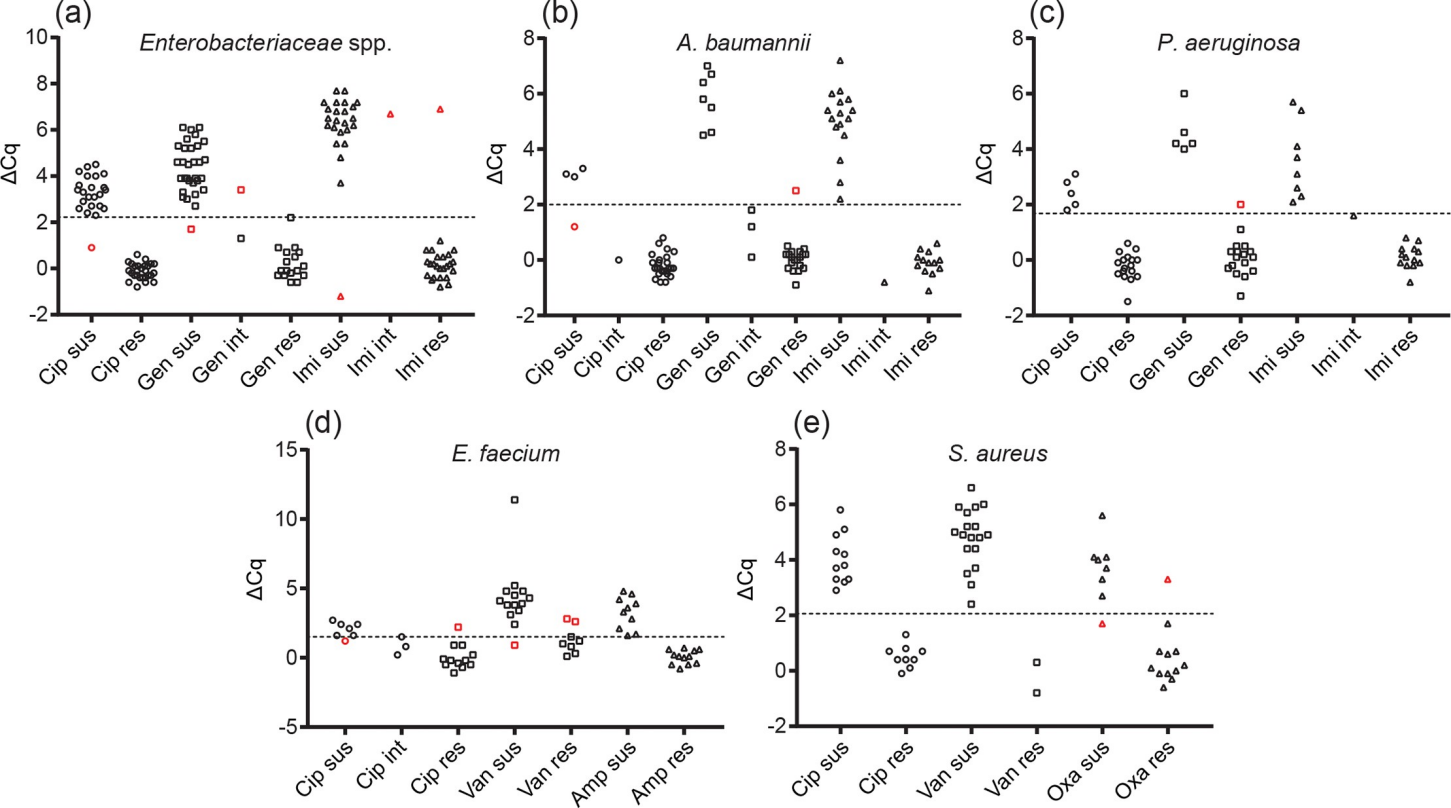
Distribution of ΔCq values for tested isolates. The ΔCq values for all isolates are shown, separated by antibiotic and susceptibility as determined by the reference method (BMD), for (a) *Enterobacteriaceae* spp., (b) *A*. *baumannii*, (c) *P*. *aeruginosa*, (d) *E*. *faecium*, and (e) *S*. *aureus*. Isolate-antibiotic combinations that resulted in minor, major, or very major errors are colored red. Sus, susceptible; int, intermediate; res, resistant; Cip, ciprofloxacin; Gen, gentamicin; Imi, imipenem; Van, vancomycin; Amp, ampicillin; Oxa, oxacillin.

With the optimized assay conditions, we tested a total of 432 antibiotic-species combinations. We found an overall categorical agreement of 96.3%, with a major error rate of 3.7% and a very major error rate of 3.1% (Table 2, Fig 1) compared to broth microdilution (BMD). Major and very major error rates were calculated as the numbers of false resistant or false susceptible results divided by the numbers of true susceptible or true resistant isolates, respectively. The numbers of true susceptible and true resistant isolates were used in the denominator rather than the total number of samples (the latter method is occasionally reported in AST assay evaluation but provides misleadingly low error rates). Calculations were performed in this manner to accurately reflect the high error rates resulting from even a single error when a small population of either resistant or susceptible isolates are used for assay evaluation. We treated a susceptible result from the RAMAST assay for an intermediate resistance strain as a minor error, with only 2 minor errors from 12 intermediate strains, as the assay produces a binary result limiting the application for intermediate resistance classification. Retesting major or very major errors from specific antibiotic-strain combinations resulted in 5 of 14 producing correct results upon re-inspection (Table F in S1 File). These conflicting data could be due to operator error, failure in an extraction column, or erroneous qPCR readings. In the future, automation of the sample preparation procedure would reduce the potential for operator error while also minimizing hands-on time and decreasing assay time. A possibility for repeat errors on retesting may be that these strains simply behave differently than other strains of the same species, possibly due to different mechanisms of resistance or genetic variability in other parts of the genome.

**Table 2:** Categorical agreement and errors for the tested isolates.

Antibiotic	Isolates resistant by BMD		Isolates susceptible by BMD		Isolates intermediate by BMD		Total replicates	Very major error rate	Major error rate	Number of minor errors	Categorical agreement
	Susceptible by qPCR	Resistant by qPCR	Susceptible by qPCR	Resistant by qPCR	Susceptible by qPCR	Resistant by qPCR					
Ciprofloxacin	1	89	47	3	0	4	144	1.1%	6.0%	0	97.2%
Gentamicin	1	52	42	1	1	4	102	3.7%	2.3%	1	96.1%
Imipenem	1	50	47	1	1	2	102	2.0%	2.1%	1	97.1%
Oxacillin	1	11	7	1	0	0	20	8.3%	12.5%	0	90.0%
Ampicillin	0	12	10	0	0	0	22	0.0%	0.0%	0	100.0%
Vancomycin	2	8	31	1	0	0	42	20.0%	3.1%	0	92.9%
Overall	7	222	184	7	2	10	432	3.1%	3.7%	2	96.3%

The RAMAST assay generally performed well when parsing results by species and antibiotic with *Enterobacteriaceae* spp., *A*. *baumannii*, *P*. *aeruginosa*, and *S*. *aureus* (96–99% categorical agreement), but resulted in a larger number of errors with *E*. *faecium* (92.4% categorical agreement, with 2 major errors and 3 very major errors) (Fig 1, Table G in S1 File). The combination of vancomycin with *E*. *faecium* proved particularly challenging, with two very major errors and one major error at the optimized antibiotic concentration (25% very major error rate, 7% major error rate). Moreover, repeating the assay on the erroneously called isolates with vancomycin resulted in replication of both very major errors (Table F in S1 File). Attempts to further optimize the assay to reduce the very major error rate through the use of lower vancomycin concentrations resulted in unacceptably large numbers of major errors (Table H in S1 File). We also obtained high very major error rates with ciprofloxacin in *E*. *faecium* and oxacillin in *S*. *aureus* (both 8.3% very major error rates). The relatively small sample sizes for these antibiotic-species combinations may have contributed to these high very major error rates. A longer antibiotic incubation or other assay optimization is likely necessarily to achieve a more acceptable level of accuracy. Similarly, ciprofloxacin and *A*. *baumannii* yielded a major error rate of 25%; but, again, the sample set was small with only 4 susceptible strains.

Several similar studies reported qPCR assays for AST but, to the best of our knowledge, only the study by Beuving et al. used samples from blood culture bottles [19]. As discussed above, the presence of blood cells and other serum components introduces complexities beyond what might be expected for performing qPCR on pure culture or urine samples. Compared to Beuving et al., we reduced the antibiotic incubation time from 6 h to 1 or 2 h, depending on the antibiotic, by using a richer media and minimally diluted positive blood culture soon after positivity. The qPCR cycling conditions used here with the BactQuant primers and probe were also significantly faster, enabling an overall reduction in assay turn-around-time from 9 h to 3 or 4 h (depending on antibiotic).

### Bacterial identification through amplicon sequencing

As 16S sequence data is often used for definitive bacterial identification for infrequently isolated pathogens, we explored if the RAMAST assay amplicon could be repurposed for genus identification given the broad coverage of the primer set. As mentioned, the BactQuant primers target variable regions V3 and V4 of the 16S. Stringent analysis of ribosomal sequences from medically relevant pathogens suggest the V3 region is suitable for distinguishing all bacterial regions down to the genus level except for closely related *Enterobacteriaceae* [29]. Using next-generation sequencing, we batch sequenced a subset of 29 strains used in this study, covering 17 different species, to test whether the RAMAST assay amplicon could be used in this fashion. These experiments resulted in correct genus level concordance for all species (Table 3), with the exception of *Klebsiella oxytoca*, through read mapping to a medically relevant 16S ribosomal database as described in Stefan et al. [26]. In the exception of *Klebsiella*, a higher percentage of reads mapped to *Enterobacter* for *K*. *oxytoca*; however, *Klebsiella* had the second highest percentage of mapped reads and the two genera are both closely related *Enterobacteriaceae*. Although this method for performing species identification is slower than other available methods (i.e., MALDI-TOF, biochemical), 16S amplicon sequencing is a useful tool for infrequently isolated bacteria that may be misidentified by other methods. Although relatively short, the amplicon from the RAMAST assay provided sufficiently long sequences through variable regions of the 16S gene for differentiation and was adequate for sequencing by Sanger or next generation sequencing techniques.

**Table 3:** Read mapping of sequenced qPCR amplicons.

	Highest% mapped		2nd Highest% mapped*	
Input	Genus	% Mapped Reads	Genus	% Mapped Reads
*E*. *coli*	*Escherichia/Shigella*	98.03	*-*	*-*
*E*. *coli*	*Escherichia/Shigella*	92.59	*Vibrio*	3.10
*E*. *coli*	*Escherichia/Shigella*	94.96	*Vibrio*	1.76
*K*. *pneumoniae*	*Klebsiella*	77.99	*Enterobacter*	11.92
*K*. *pneumoniae*	*Klebsiella*	82.78	*Enterobacter*	8.34
*K*. *pneumoniae*	*Klebsiella*	79.48	*Enterobacter*	10.28
*A*. *baumannii*	*Acinetobacter*	99.95	*-*	*-*
*A*. *baumannii*	*Acinetobacter*	99.78	*-*	*-*
*A*. *baumannii*	*Acinetobacter*	99.09	*-*	*-*
*S*. *aureus*	*Staphylococcus*	99.70	*-*	*-*
*S*. *aureus*	*Staphylococcus*	99.17	*-*	*-*
*S*. *aureus*	*Staphylococcus*	99.93	*-*	*-*
*E*. *faecium*	*Enterococcus*	99.04	*-*	*-*
*E*. *faecium*	*Enterococcus*	98.32	*-*	*-*
*E*. *faecium*	*Enterococcus*	98.86	*-*	*-*
*P*. *aeruginosa*	*Pseudomonas*	99.89	*-*	*-*
*P*. *aeruginosa*	*Pseudomonas*	99.16	*-*	*-*
*P*. *aeruginosa*	*Pseudomonas*	99.83	*-*	*-*
*Enterobacter cloacae*	*Enterobacter*	85.49	*-*	*-*
*Enterobacter aerogenes*	*Enterobacter*	66.07	*Raoultella*	10.15
*Citrobacter freundii*	*Citrobacter*	88.76	*Kluyvera*	5.67
*Citrobacter koseri*	*Citrobacter*	75.66	*Salmonella*	15.00
*Proteus stuartii*	*Proteus*	86.64	*Vibrio*	5.74
*Serratia marcescens*	*Serratia*	77.69	*Vibrio*	21.15
*Klebsiella oxytoca*	*Enterobacter*	58.39	*Klebsiella*	25.82
*Proteus mirabilis*	*Proteus*	99.90	*-*	*-*
*Shigella sonnei*	*Escherichia/Shigella*	99.25	*-*	*-*
*Salmonella typhimurium*	*Salmonella*	98.34	*-*	*-*
*Salmonella senftenberg*	*Salmonella*	94.91	*Enterobacter*	3.76

* Only samples with less than 95% of reads mapped have a second genus shown

## Conclusion

Here, we showed that use of the RAMAST assay for relative DNA quantification after an antibiotic incubation could be used to generate phenotypic antibiotic susceptibility results in as little as 3 to 4 hours from positive blood culture. The use of the universal BactQuant 16S amplification system simplified the assay, requiring only a single set of primers and probes for testing of all bacterial pathogens. Further improvement of this assay could be achieved through the application of recently reported thermo-cold lysis for DNA extraction, potentially decreasing hands-on time and operator error [21]. Overall, our assay showed high categorical agreement at 96.3% while representing a significant decrease in total assay time compared to a similar qPCR AST assay from blood culture.

## Supporting information

S1 File This file contains strain information, raw MIC and ΔCq value data, detailed result statistics, and additional figures. (PDF)
