# Supplementary material for: Rapid antibiotic susceptibility testing from blood culture bottles with species agnostic real-time polymerase chain reaction
Source: PLoS One. 2018 Dec 13;13(12):e0209042. doi: 10.1371/journal.pone.0209042 (PMC6292663; doi:10.1371/journal.pone.0209042)
Supplement: S1 File — (PDF) [file pone.0209042.s001.pdf]

Table A. *Enterobacteriaceae* spp. strain information.

Species	Strain	Origin	Ciprofloxacin			Gentamicin			Imipenem		
			BMD (MIC; int*)	qPCR assay (ΔCq)	qPCR assay (int*)	BMD (MIC; int*)	qPCR assay (ΔCq)	qPCR assay (int*)	BMD (MIC; int*)	qPCR assay (ΔCq)	qPCR assay (int*)
Escherichia coli	AR0001	CDC	> 32; R	0.2	R	2; S	3.1	S	4; R	-0.5	R
Enterobacter cloacae	AR0002	CDC	16; R	-0.4	R	8; I	3.4	S	32; R	0.2	R
Klebsiella pneumoniae	AR0003	CDC	16; R	0.0	R	4; S	3.3	S	8; R	0.1	R
Klebsiella pneumoniae	AR0004	CDC	16; R	-0.6	R	1; S	3.9	S	32; R	-0.4	R
Klebsiella pneumoniae	AR0005	CDC	32; R	0.2	R	2; S	2.7	S	16; R	-0.8	R
Escherichia coli	AR0006	CDC	> 32; R	-0.3	R	> 64; R	-0.3	R	8; R	0.3	R
Enterobacter aerogenes	AR0007	CDC	32; R	-0.3	R	1; S	6.1	S	0.5; S	7.7	S
Enterobacter cloacae	AR0008	CDC	16; R	0.1	R	≤ 0.5; S	4.5	S	1; S	5.9	S
Enterobacter aerogenes	AR0009	CDC	≤ 0.25; S	3.4	S	1; S	4.6	S	4; R	0.8	R
Klebsiella pneumoniae	AR0010	CDC	≤ 0.25; S	2.3	S	4; S	3.2	S	1; S	5.4	S
Escherichia coli	AR0011	CDC	> 32; R	0.2	R	64; R	-0.2	R	≤ 0.25; S	6.8	S
Klebsiella pneumoniae	AR0012	CDC	16; R	0.1	R	32; R	2.2	R	≤ 0.25; S	6.4	S
Escherichia coli	AR0013	CDC	≤ 0.25; S	3.6	S	> 64; R	-0.3	R	≤ 0.25; S	7.2	S
Escherichia coli	AR0014	CDC	32; R	-0.4	R	64; R	-0.1	R	≤ 0.25; S	6.1	S
Escherichia coli	AR0015	CDC	> 32; R	-0.1	R	> 64; R	0.9	R	≤ 0.25; S	6.9	S
Klebsiella pneumoniae	AR0016	CDC	≤ 0.25; S	2.7	S	≤ 0.5; S	6.1	S	≤ 0.25; S	7.7	S
Escherichia coli	AR0017	CDC	≤ 0.25; S	3.1	S	1; S	1.7	R	1; S	3.7	S
Enterobacter aerogenes	AR0018	CDC	≤ 0.25; S	4.4	S	1; S	5.8	S	1; S	6.3	S
Escherichia coli	AR0019	CDC	> 32; R	-0.2	R	≤ 0.5; S	3.9	S	≤ 0.25; S	6.2	S
Escherichia coli	AR0020	CDC	16; R	-0.3	R	1; S	6.0	S	1; S	7.2	S
Citrobacter freundii	AR0021	CDC	≤ 0.25; S	2.6	S	1; S	5.3	S	1; S	6.5	S
Citrobacter freundii	AR0022	CDC	≤ 0.25; S	3.5	S	≤ 0.5; S	4.6	S	1; S	5.4	S
Citrobacter freundii	AR0023	CDC	≤ 0.25; S	2.6	S	≤ 0.5; S	5.3	S	1; S	6.2	S
Citrobacter koseri	AR0024	CDC	≤ 0.25; S	3.2	S	1; S	5.2	S	≤ 0.25; S	7.0	S
Citrobacter koseri	AR0025	CDC	≤ 0.25; S	4.5	S	64; R	0.7	R	≤ 0.25; S	7.2	S
Providencia stuartii	AR0026	CDC	≤ 0.25; S	3.4	S	1; S	3.9	S	0.5; S	-1.2	R
Serratia marcescens	AR0027	CDC	≤ 0.25; S	0.9	R	≤ 0.5; S	3.4	S	4; R	-0.4	R
Klebsiella oxytoca	AR0028	CDC	≤ 0.25; S	3.5	S	≤ 0.5; S	4.6	S	≤ 0.25; S	7.2	S
Proteus mirabilis	AR0029	CDC	≤ 0.25; S	4.2	S	1; S	3.7	S	8; R	-0.4	R
Shigella sonnei	AR0030	CDC	≤ 0.25; S	4.1	S	1; S	4.6	S	≤ 0.25; S	6.8	S
Salmonella typhimurium	AR0031	CDC	≤ 0.25; S	3.1	S	1; S	3.8	S	≤ 0.25; S	4.8	S
Klebsiella pneumoniae	AR0112	CDC	32; R	-0.3	R	16; R	-0.1	R	4; R	0.0	R
Klebsiella pneumoniae	AR0113	CDC	> 32; R	0.1	R	>64; R	-0.3	R	16; R	-0.3	R
Escherichia coli	AR0114	CDC	16; R	-0.1	R	4; S	3.0	S	> 32; R	0.3	R
Klebsiella pneumoniae	AR0115	CDC	32; R	0.1	R	8; I	1.3	R	> 32; R	-0.1	R
Citrobacter freundii	AR0116	CDC	8; R	0.4	R	16; R	0.5	R	8; R	0.6	R
Klebsiella pneumoniae	AR0117	CDC	4; R	-0.2	R	16; R	0.3	R	16; R	0.5	R

Escherichia coli	AR0118	CDC	32; R	0.6	R	> 64; R	-0.6	R	1; S	6.5	S
Escherichia coli	AR0119	CDC	> 32; R	-0.4	R	> 64; R	-0.1	R	0.5; S	6.0	S
Klebsiella pneumoniae	AR0120	CDC	32; R	-0.6	R	64; R	0.1	R	16; R	0.8	R
Serratia marcescens	AR0121	CDC	1; S	2.9	S	≤ 0.5; S	3.9	S	> 32; R	0.2	R
Serratia marcescens	AR0122	CDC	≤ 0.25; S	4.0	S	≤ 0.5; S	5.6	S	> 32; R	0.2	R
Serratia marcescens	AR0123	CDC	≤ 0.25; S	2.7	S	≤ 0.5; S	5.5	S	> 32; R	1.2	R
Serratia marcescens	AR0124	CDC	≤ 0.25; S	2.4	S	1; S	3.8	S	> 32; R	0.8	R
Klebsiella pneumoniae	AR0125	CDC	> 32; R	-0.8	R	> 64; R	-0.6	R	16; R	-0.7	R
Klebsiella pneumoniae	AR0126	CDC	≤ 0.25; S	3.3	S	≤ 0.5; S	4.7	S	8; R	0.0	R
Salmonella senftenberg	AR0127	CDC	16; R	0.3	R	> 64; R	0.9	R	2; I	6.7	S
Escherichia coli	AR0128	CDC	32; R	-0.6	R	> 64; R	0.7	R	8; R	6.9	S
Klebsiella pneumoniae	AR0129	CDC	> 32; R	-0.1	R	2; S	5.2	S	8; R	0.5	R
Serratia marcescens	AR0130	CDC	≤ 0.25; S	4.0	S	1; S	3.9	S	> 32; R	0.1	R

* int = categorical interpretation

Table B. *A. baumannii* strain information.

			Ciprofloxacin			Gentamicin			Imipenem		
Species	Strain	Origin	BMD (MIC; int*)	qPCR assay (ΔCq)	qPCR assay (int*)	BMD (MIC; int*)	qPCR assay (ΔCq)	qPCR assay (int*)	BMD (MIC; int*)	qPCR assay (ΔCq)	qPCR assay (int*)
A. baumannii	AR0033	CDC	32; R	-0.3	R	> 64; R	-0.4	R	2; S	2.8	S
A. baumannii	AR0035	CDC	32; R	-0.3	R	8; I	0.1	R	> 32; R	-0.2	R
A. baumannii	AR0036	CDC	> 32; R	0.3	R	8; I	1.2	R	32; R	0.4	R
A. baumannii	AR0037	CDC	32; R	-0.3	R	> 64; R	-0.3	R	2; S	3.6	S
A. baumannii	AR0045	CDC	16; R	-0.3	R	> 64; R	0.4	R	8; R	-0.1	R
A. baumannii	AR0052	CDC	4; R	0.8	R	> 64; R	0.2	R	8; R	0.1	R
A. baumannii	AR0056	CDC	32; R	-0.7	R	> 64; R	-0.9	R	32; R	-1.1	R
A. baumannii	AR0063	CDC	32; R	-0.8	R	2; S	6.4	S	8; R	0.0	R
A. baumannii	AR0070	CDC	4; R	-0.1	R	64; R	-0.2	R	8; R	0.6	R
A. baumannii	AR0078	CDC	> 32; R	-0.1	R	> 64 ; R	0.3	R	16; R	0.3	R
A. baumannii	AR0083	CDC	8; R	-0.1	R	> 64; R	0.2	R	32; R	0.0	R
A. baumannii	AR0088	CDC	16; R	-0.4	R	> 64; R	0.2	R	2; S	2.2	S
A. baumannii	AR0101	CDC	32; R	-0.6	R	4; S	4.5	S	> 32; R	-0.3	R
A. baumannii	AR0102	CDC	16; R	-0.4	R	> 64; R	-0.4	R	1; S	4.5	S
A. baumannii	NR-13380	BEI	16; R	-0.3	R	> 64; R	0.2	R	0.5; S	5.4	S
A. baumannii	NR-13383	BEI	> 32; R	-0.5	R	> 64; R	0.1	R	4; I	-0.8	R
A. baumannii	NR-17780	BEI	16; R	0.6	R	>64; R	0.5	R	0.5; S	6.1	S
A. baumannii	NR-17781	BEI	2; I	0.0	R	> 64; R	0.3	R	1; S	5.3	S
A. baumannii	NR-17782	BEI	32; R	0.1	R	> 64; R	0.0	R	0.5; S	4.9	S
A. baumannii	NR-17784	BEI	> 32; R	-0.5	R	> 64; R	-0.1	R	0.5; S	7.2	S
A. baumannii	NR-13379	BEI	32; R	-0.8	R	> 64; R	0.0	R	16; R	-0.1	R

A. baumannii	NR-13378	BEI	16; R	-0.3	R	8; I	1.8	R	> 32; R	-0.4	R
A. baumannii	NR-17786	BEI	16; R	0.0	R	> 64; R	-0.3	R	1; S	4.8	S
A. baumannii	NR-17778	BEI	≤ 0.25; S	1.2	R	1; S	7.0	S	≤ 0.25; S	5.1	S
A. baumannii	NR-17787	BEI	32; R	-0.4	R	> 64; R	0.1	R	≤ 0.25; S	5.7	S
A. baumannii	NR-13375	BEI	> 32; R	0.2	R	32; R	2.5	S	16; R	-0.5	R
A. baumannii	CNH111	FDA	32; R	0.4	R	2; S	4.6	S	≤ 0.25; S	5.4	S
A. baumannii	CNH151	FDA	≤ 0.25; S	3.0	S	1; S	5.8	S	≤ 0.25; S	5.1	S
A. baumannii	CNH160	FDA	≤ 0.25; S	3.3	S	4; S	6.7	S	≤ 0.25; S	5.8	S
A. baumannii	CNH640	FDA	≤ 0.25; S	3.1	S	2; S	5.5	S	≤ 0.25; S	6.0	S

* int = categorical interpretation

Table C. *P. aeruginosa* strain information.

			Ciprofloxacin			Gentamicin			Imipenem		
Species	Strain	Origin	BMD (MIC; int*)	qPCR assay (ΔCq)	qPCR assay (int*)	BMD (MIC; int*)	qPCR assay (ΔCq)	qPCR assay (int*)	BMD (MIC; int*)	qPCR assay (ΔCq)	qPCR assay (int*)
P. aeruginosa	AR0054	CDC	8; R	-0.1	R	> 64; R	-0.4	R	> 32; R	0.4	R
P. aeruginosa	AR0064	CDC	8; R	0.3	R	1; S	4.2	S	> 32; R	0.1	R
P. aeruginosa	AR0090	CDC	16; R	-0.5	R	> 64; R	0.3	R	> 32; R	0.3	R
P. aeruginosa	AR0092	CDC	32; R	-1.5	R	> 64; R	-0.5	R	> 32; R	-0.1	R
P. aeruginosa	AR0094	CDC	16; R	-0.7	R	16; R	0.1	R	32; R	0.2	R
P. aeruginosa	AR0095	CDC	16; R	0.0	R	0.5; S	4.0	S	16; R	0.8	R
P. aeruginosa	AR0100	CDC	16; R	0.0	R	> 64; R	-0.2	R	32; R	-0.8	R
P. aeruginosa	AR0103	CDC	16; R	-0.3	R	> 64; R	-1.3	R	> 32; R	-0.2	R
P. aeruginosa	AR0105	CDC	> 32; R	-0.4	R	> 64; R	0.1	R	1; S	3.7	S
P. aeruginosa	AR0108	CDC	16; R	-0.4	R	> 64; R	-0.3	R	> 32; R	0.4	R
P. aeruginosa	AR0110	CDC	16; R	0.6	R	> 64; R	0.5	R	> 32; R	0.7	R
P. aeruginosa	AR0111	CDC	8; R	-0.6	R	> 64; R	-0.1	R	> 32; R	0.0	R
P. aeruginosa	105898	USAMRIID	8; R	0.1	R	> 64; R	0.3	R	0.5; S	2.6	S
P. aeruginosa	105819	USAMRIID	4; R	0.4	R	> 64; R	0.5	R	8; R	-0.1	R
P. aeruginosa	CNH770	FDA	≤ 0.25; S	2.4	S	≤ 0.5; S	4.2	S	1; S	4.1	S
P. aeruginosa	105756	USAMRIID	4; R	0.0	R	> 64; R	0.3	R	4; I	1.6	R
P. aeruginosa	105857	USAMRIID	16; R	-0.2	R	32; R	2.0	S	> 32; R	-0.2	R
P. aeruginosa	000006	USAMRIID	32; R	-0.6	R	16; R	1.1	R	1; S	2.1	S
P. aeruginosa	CNH363	FDA	≤ 0.25; S	3.1	S	≤ 0.5; S	6.0	S	1; S	5.7	S
P. aeruginosa	NR-31041	BEI	≤ 0.25; S	2.8	S	64; R	-0.6	R	2; S	2.3	S
P. aeruginosa	NR-50573	BEI	≤ 0.25; S	2.0	S	≤ 0.5; S	4.6	S	0.5; S	5.4	S
P. aeruginosa	NR-48928	BEI	≤ 0.25; S	1.8	S	> 64; R	0.2	R	1; S	3.1	S

* int = categorical interpretation

Table D. *E. faecium* strain information.

Species	Strain	Origin	Ciprofloxacin			Vancomycin			Ampicillin		
			BMD (MIC; int*)	qPCR assay (ΔCq)	qPCR assay (int*)	BMD (MIC; int*)	qPCR assay (ΔCq)	qPCR assay (int*)	BMD (MIC; int*)	qPCR assay (ΔCq)	qPCR assay (int*)
E. faecium	NR-32065	BEI	4; R	0.9	R	> 32; R	0.1	R	4; S	2.1	S
E. faecium	NR-32094	BEI	> 32; R	0.2	R	32; R	0.3	R	32; R	0.0	R
E. faecium	HM-463	BEI	32; R	-0.5	R	0.5; S	2.4	S	16; R	0.7	R
E. faecium	NR-32053	BEI	≤ 0.25; S	2.7	S	> 32; R	1.5	R	2; S	3.3	S
E. faecium	NR-31967	BEI	8; R	-0.1	R	1; S	5.2	S	> 64; R	0.6	R
E. faecium	NR-31916	BEI	1; S	1.6	S	> 32; R	2.6	S	8; S	2.8	S
E. faecium	NR-31937	BEI	2; I	1.5	R	0.5; S	0.9	R	≤ 0.5; S	1.7	S
E. faecium	NR-28979	BEI	2; I	0.2	R	1; S	3.1	S	64; R	-0.5	R
E. faecium	NR-28982	BEI	> 32; R	-0.7	R	1; S	3.8	S	64; R	0.2	R
E. faecium	HM-204	BEI	1; S	1.2	R	1; S	4.8	S	2; S	3.9	S
E. faecium	NR-31963	BEI	4; R	0.9	R	1; S	4.8	S	16; R	-0.5	R
E. faecium	NR-31954	BEI	16; R	2.2	S	1; S	3.4	S	2; S	4.2	S
E. faecium	NR-28976	BEI	≤ 0.25; S	2.4	S	1; S	4.1	S	2; S	4.8	S
E. faecium	NR-31955	BEI	> 32; R	-1.1	R	0.5; S	4.5	S	32; R	0.1	R
E. faecium	NR-31912	BEI	32; R	-0.2	R	> 32; R	1.2	R	32; R	-0.4	R
E. faecium	NR-31913	BEI	1; S	2.1	S	0.5; S	11.4	S	8; S	4.6	S
E. faecium	NR-32052	BEI	0.5; S	1.6	S	> 32; R	0.8	R	4; S	1.6	S
E. faecium	NR-32054	BEI	1; S	2.4	S	> 32; R	1.0	R	4; S	3.6	S
E. faecium	GRIM718	USAMRIID	> 32; R	-0.5	R	> 32; R	2.8	S	64; R	0.6	R
E. faecium	GRIM725	USAMRIID	2; I	0.8	R	1; S	3.9	S	> 64; R	-0.8	R
E. faecium	GRIM726	USAMRIID	> 32; R	-0.2	R	1; S	4.3	S	64; R	0.5	R
E. faecium	10857	USAMRIID	> 32; R	-0.4	R	1; S	3.8	S	64; R	0.1	R

* int = categorical interpretation

Table E. *S. aureus* strain information.

Species	Strain	Origin	Ciprofloxacin			Vancomycin			Oxacillin		
			BMD (MIC; int*)	qPCR assay (ΔCq)	qPCR assay (int*)	BMD (MIC; int*)	qPCR assay (ΔCq)	qPCR assay (int*)	BMD (MIC; int*)	qPCR assay (ΔCq)	qPCR assay (int*)
S. aureus	ATCC BAA-38	ATCC	≤ 0.25; S	3.3	S	1; S	3.7	S	32; R	0.1	R
S. aureus	ATCC BAA-39	ATCC	4; R	0.7	R	2; S	4.9	S	32; R	0	R
S. aureus	ATCC BAA-40	ATCC	≤ 0.25; S	2.9	S	2; S	5	S	> 32; R	-0.3	R
S. aureus	ATCC BAA-41	ATCC	8; R	0.4	R	2; S	5.7	S	> 32; R	1.7	R
S. aureus	ATCC BAA-43	ATCC	32; R	0.4	R	1; S	4.4	S	> 32; R	0.2	R
S. aureus	ATCC BAA-44	ATCC	4; R	0.7	R	2; S	3.1	S	> 32; R	0.7	R
S. aureus	GRIM48	USAMRIID	≤ 0.25; S	3.3	S	1; S	3.5	S	≤ 0.25; S	3.3	S

S. aureus	FDAARGOS_159	FDA	≤ 0.25; S	3.7	S	2; S	4.9	S	≤ 0.25; S	4.1	S
S. aureus	FDAARGOS_169	FDA	1; S	3.8	S	1; S	4.4	S	16; R	-0.1	R
S. aureus	ATCC 700698	ATCC	32; R	-0.1	R	2; S	2.4	S	> 32; R	-0.6	R
S. aureus	FDAARGOS_135	FDA	4; R	0.8	R	1; S	5.2	S	8; R	3.3	S
S. aureus	FDAARGOS_140	FDA	4; R	0.4	R	1; S	5.9	S	8; R	0.6	R
S. aureus	71080	BEI	32; R	1.3	R	> 32; R	0.3	R	> 32; R	0.7	R
S. aureus	ATCC 25923	ATCC	≤ 0.25; S	5.8	S	2; S	4.8	S	≤ 0.25; S	4	S
S. aureus	ATCC 29213	ATCC	≤ 0.25; S	4.3	S	1; S	6	S	≤ 0.25; S	3.7	S
S. aureus	ATCC 12600	ATCC	≤ 0.25; S	5.1	S	1; S	5.9	S	≤ 0.25; S	2.7	S
S. aureus	ATCC 13565	ATCC	≤ 0.25; S	4.9	S	1; S	4.8	S	≤ 0.25; S	4.1	S
S. aureus	ATCC 14458	ATCC	≤ 0.25; S	3.2	S	1; S	5.2	S	0.5; S	1.7	R
S. aureus	ATCC 8095	ATCC	≤ 0.25; S	4.2	S	1; S	6.6	S	≤ 0.25; S	5.6	S
S. aureus	880 (BR-VRSA)	BEI	16; R	0.1	R	16; R	-0.8	R	> 32; R	-0.1	R

* int = categorical interpretation

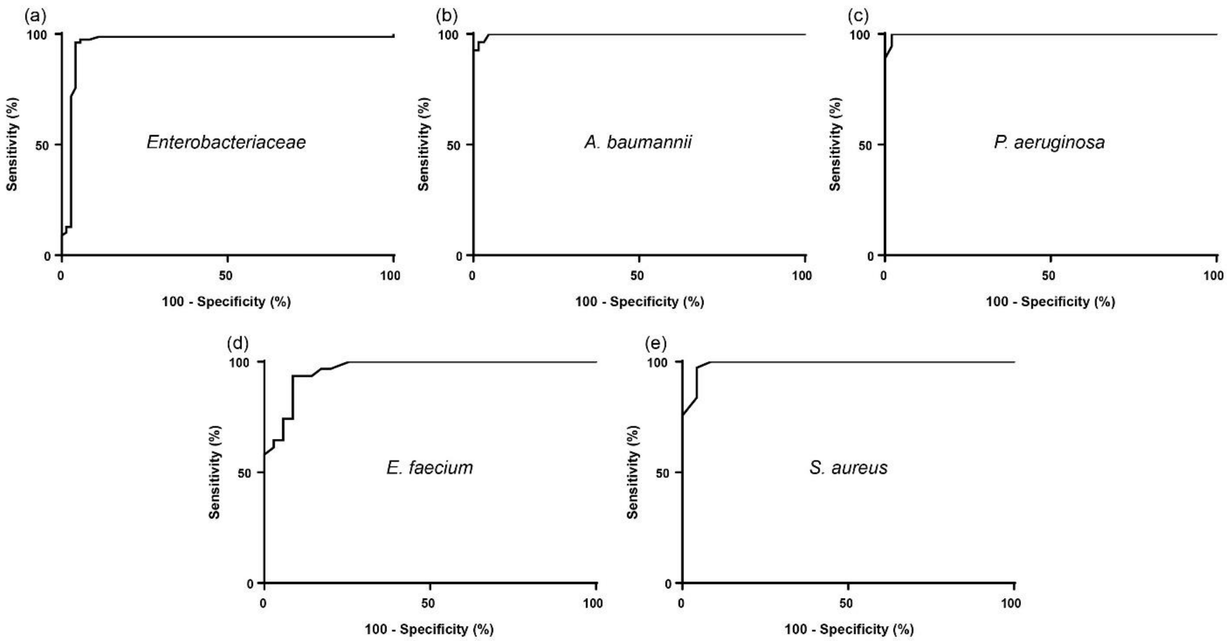

Figure A. ROC curves derived from the experimental ΔCq values. ROC curves were generated such that resistance was considered the "control". Sensitivity is then defined as the ability of the assay to call true susceptibility and is a measure of the major error rate. Specificity is defined as the ability of the assay to call true resistance and is a measure of the very major error rate. Cutoff values were selected to maximize the likelihood ratio for a correct susceptibility call. (a) *Enterobacteriaceae*, (b) *A. baumannii*, (c) *P. aeruginosa*, (d) *E. faecium*, (e) *S. aureus*.

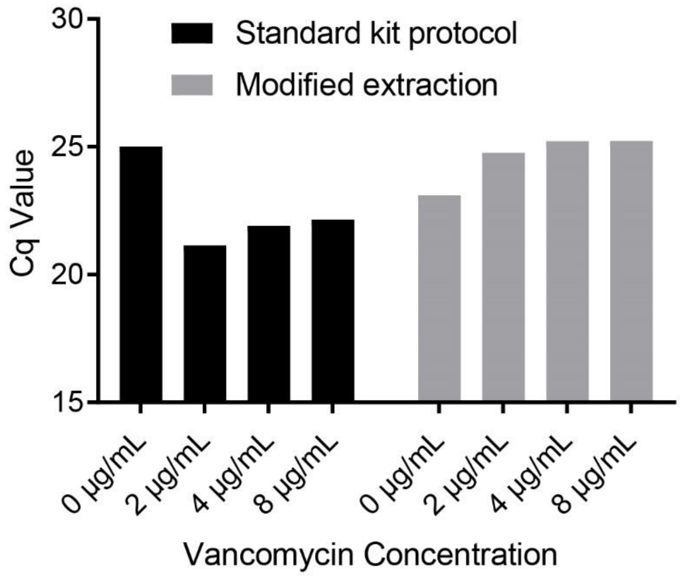

Figure B. Example of insufficient bacterial lysis. The RAMAST assay was performed on cultures of *S. aureus* ATCC 25923, utilizing two different DNA extraction methods. For the standard kit protocol, DNA was extracted using an automated EZ1 instrument (Qiagen, Hilden, Germany) following the manufacturer's protocol with a DNA tissue kit and a DNA bacteria card. The modified extraction protocol is described in the materials and methods. Using the standard kit protocol resulted in inconsistent lysis / extraction for high loads of Gram positive bacteria, a situation that is often the case for no-antibiotic samples and for resistant strains.

Table F. Retesting of strain-antibiotic combinations originally resulting in errors.

Species	Strain	Antibiotic	BMD (MIC; int*)	qPCR assay (ΔCq)	qPCR assay (int*)
E. coli	AR0017	Gentamicin	1; S	1.5	R
P. stuartii	AR0026	Imipenem	0.5; S	-0.8	R
S. marcescens	AR0027	Ciprofloxacin	≤ 0.25; S	1.5	R
E. coli	AR0128	Imipenem	8; R	5.6	S
A. baumannii	NR-17778	Ciprofloxacin	≤ 0.25; S	-1.7	R
A. baumannii	NR-13375	Gentamicin	32; R	-0.1	R
P. aeruginosa	105857	Gentamicin	32; R	0.3	R
E. faecium	NR-31916	Vancomycin	> 32; R	5.4	S
E. faecium	NR-31937	Vancomycin	0.5; S	2.7	S
E. faecium	HM-204	Ciprofloxacin	1; S	3.5	S
E. faecium	NR-31954	Ciprofloxacin	16; R	2	S
E. faecium	GRIM718	Vancomycin	> 32; R	2.6	S
S. aureus	ATCC 14458	Oxacillin	0.5; S	0.6	R
S. aureus	FDAARGOS_135	Oxacillin	8; R	-1.2	R

* int = categorical interpretation

Table G. Categorical agreement and errors for each strain and antibiotic.

	Isolates resistant by BMD		Isolates susceptible by BMD		Isolates intermediate by BMD		Total replicates	Very major error rate	Major error rate	Number of minor errors	Categorical agreement
	Susceptible by BQ	Resistant by BQ	Susceptible by BQ	Resistant by BQ	Susceptible by BQ	Resistant by BQ					
Enterobacteriaceae											
Ciprofloxacin	0	27	22	1	0	0	50	0.0%	4.3%	0	98.0%
Gentamicin	0	17	30	1	1	1	50	0.0%	3.2%	1	96.0%
Imipenem	1	24	23	1	1	0	50	4.0%	4.2%	1	94.0%
Overall	1	68	75	3	2	1	150	1.4%	3.8%	2	96.0%
A. baumannii											
Ciprofloxacin	0	25	3	1	0	1	30	0.0%	25.0%	0	96.7%
Gentamicin	1	19	7	0	0	3	30	5.0%	0.0%	0	96.7%
Imipenem	0	13	16	0	0	1	30	0.0%	0.0%	0	100.0%
							0				
Overall	1	57	26	1	0	5	90	1.7%	3.7%	0	97.8%
P. aeruginosa											
Ciprofloxacin	0	17	5	0	0	0	22	0.0%	0.0%	0	100.0%
Gentamicin	1	16	5	0	0	0	22	5.9%	0.0%	0	95.5%
Imipenem	0	13	8	0	0	1	22	0.0%	0.0%	0	100.0%
Overall	1	46	18	0	0	1	66	2.1%	0.0%	0	98.5%
E. faecium											
Ciprofloxacin	1	11	6	1	0	3	22	8.3%	14.3%	0	90.9%
Vancomycin	2	6	13	1	0	0	22	25.0%	7.1%	0	86.4%
Ampicillin	0	12	10	0	0	0	22	0.0%	0.0%	0	100.0%
Overall	3	29	29	2	0	3	66	9.4%	6.5%	0	92.4%
S. aureus											
Ciprofloxacin	0	9	11	0	0	0	20	0.0%	0.0%	0	100.0%
Vancomycin	0	2	18	0	0	0	20	0.0%	0.0%	0	100.0%
Oxacillin	1	11	7	1	0	0	20	8.3%	12.5%	0	90.0%
Overall	1	22	36	1	0	0	60	4.3%	2.7%	0	96.7%

Table H. Vancomycin concentration optimization for *E. faecium*

Species	Strain	Vancomycin						
		BMD	qPCR 0.25 µg/mL	qPCR 0.5 µg/mL	qPCR 1 µg/mL	qPCR 2 µg/mL	qPCR 4 µg/mL	qPCR 8 µg/mL
E. faecium	NR-32065	> 32; R	R	R	R	R	S	S
E. faecium	NR-32094	32; R	R	R	R	R	R	R
E. faecium	HM-463	0.5; S	R	S	S	S	S	S
E. faecium	NR-32053	> 32; R	R	R	R	R	S	S
E. faecium	NR-31967	1; S	R	R	S	S	S	S
E. faecium	NR-31916	> 32; R	R	R	S	S	S	S
E. faecium	NR-31937	0.5; S	R	R	R	R	S	S
E. faecium	NR-28979	1; S	R	R	S	S	S	S
E. faecium	NR-28982	1; S	R	R	S	S	S	S
E. faecium	HM-204	1; S	R	R	S	S	S	S
E. faecium	NR-31963	1; S	R	R	S	S	S	S
E. faecium	NR-31954	1; S	R	R	S	S	S	S
E. faecium	NR-28976	1; S	R	S	S	S	S	S
E. faecium	NR-31955	0.5; S	R	S	S	S	S	S
E. faecium	NR-31912	> 32; R	R	R	R	R	S	S
E. faecium	NR-31913	0.5; S	R	S	S	S	S	S
E. faecium	NR-32052	> 32; R	R	R	R	R	R	S
E. faecium	NR-32054	> 32; R	R	S	R	S	S	S
E. faecium	GRIM718	> 32; R	R	R	S	S	S	S
E. faecium	GRIM725	1; S	R	S	S	S	S	S
E. faecium	GRIM726	1; S	R	S	S	S	S	S
E. faecium	10857	1; S	R	S	S	S	S	S
